# Anti-diabetic potential of *Catharanthus roseus* Linn. and its effect on the glucose transport gene (GLUT-2 and GLUT-4) in streptozotocin induced diabetic wistar rats

**DOI:** 10.1186/s12906-015-0899-6

**Published:** 2015-10-21

**Authors:** Waleed M. Al-Shaqha, Mohsin Khan, Nasir Salam, Arezki Azzi, Anis Ahmad Chaudhary

**Affiliations:** College of Medicine, Al-Imam Mohammad Ibn Saud Islamic University (IMSIU), Riyadh, 13317-7544 Kingdom of Saudi Arabia (KSA); Department of Energy and Environmental sciences, Chaudhary Devi Lal University, Sirsa, Haryana 125055 India

**Keywords:** *Catharanthus roseus*, Streptozotocin, Glucose Transporter (GLUT), Wistar Rats, Real Time Polymerase Chain Reaction

## Abstract

**Background:**

*Catharanthus roseus* is an important Ayurvedic medication in traditional medicine. It is potentially used in countries like India, South Africa, China and Malaysia for the healing of diabetes mellitus. Although, the molecular mechanisms behind this effect are yet to be exclusively explored. Due to the great antidiabetic and hyperlipidemic potential of *c. roseus*, we hypothesized that the insulin mimetic effect of ethanolic extract of *c. roseus* might add to glucose uptake through improvement in the expression of genes of the glucose transporter (GLUT) family messenger RNA (mRNA) in liver.

**Methods:**

STZ-induced diabetic rats treated by ethanolic extract of c. roseus 100 mg/kg and 200 mg/kg; and one group treated with Metformin (100 mg/kg). After final administration of treatment of 4 weeks, blood samples were collected under fasting conditions, and the body weights (BWs) were measured. Total RNA from liver was extracted with the Qiagen RNEasy Micro kit (GERMANY) as described in the manufacturer’s instructions. First-strand complementary DNA (cDNA) was synthesized at 40 °C by priming with oligo-dT12–18 (Invitrogen, USA) and using Super ScriptII reverse transcriptase according to the protocol provided by the manufacturer (Invitrogen, USA). Real-time polymerase chain reaction (PCR) amplifications for GLUT-4 (gene ID: 25139) were conducted using Light-Cycler 480 (Roche, USA) with the SyBr® I nucleic acid stain (Invitrogen, USA) according to the manufacturer's instructions. Polymerase chain reaction products of β-actin primer gene were used as an internal standard.

**Results:**

The proposed study was framed to look at the antidiabetic efficacy of ethanolic extract of *c. roseus* and an expression of GLUT-2 and GLUT-4 gene in streptozotocin induced diabetic wistar rats. The doses were administered orally at a rate of 100 and 200 mg/kg and detrain the glucose transport system in liver for 4 weeks. The observed results showed a good positive correlation between intracellular calcium and insulin release levels in isolated islets of Langerhans. The supplementation of ethanolic extract of *c. roseus* significantly amplified the expression of GLUT gene mRNA by Real Time PCR in liver of diabetic rats.

**Conclusions:**

We conclude that the observed antidiabetic effect of *c. roseus* on STZ induced diabetes was a result of complex mechanisms of GLUT gene mRNA expression. The findings are very encouraging and greatly advocate its candidature for the design of a novel herbal drug to cure deadly diabetes.

## Background

Diabetes is evolving as one of the most fatal diseases confronting humanity right behind cancer and cardiovascular diseases. Existing databases indicate its high prevalence, morbidity and mortality rate [1, 2]. About 4 % population worldwide is dying by this deadly disease and this toll is likely to swell by 5.4 % in the year 2025 [3] additionally diabetes is known to be risk factor for other diseases as well. India with its rising economy and rapidly urbanizing population is at a greater risk of this disease. The number of adults fighting with diabetes in India is projected to amplify threefold, from the existing 19.4 million in 1995 to 57.2 million in 2025. The world Health Organigation (WHO) estimated that worldwide, 346 million people have diabetes with more than 80 % of diabetics living in low and middle-income countries. The number is expected to grow to double by 2030 [4]. Recent studies on geographical and ethical influences have revealed that people of Indian origin are highly prone to diabetes. Diabetes is characterized by hyperglycemia due to an absolute or relatively deficient insulin levels [5]. Although insulin therapy is widely used for management of diabetes mellitus, its many side effects such as, insulin resistance, anorexia nervosa, brain atrophy, and fatty liver after chronic treatment makes it a risky proposition [6, 7]. Therefore, extensive research, which is still at a nascent stage, is required to find more effective and safer hypoglycemic agents. Medicinal plants are a rich source of anti-diabetic compounds and have been used for the treatment of diabetes in the form of compound drugs. Many of these plant-derived drugs against diabetes mellitus have received positive approval from WHO.

Type 2 diabetes mellitus, the most common endocrine disorder, potentially affects up to 5 % of the western population [8–10]. Patients with type 2 diabetes generally suffer both from reduced insulin secretion and from resistance to the actions of insulin. Hyperglycemic-hyperinsulinemic clamp analysis of human type 2 diabetic patients has revealed that insulin resistance in muscle is a direct consequence of the defect in glucose transport [11–13]. The principal insulin-sensitive glucose transporter in the liver and muscle is insulin-sensitive glucose transport gene (GLUT-2 and GLUT-4), which is recruited to the sarcolemma following insulin stimulation. The glucose transporter is the primary isoform in the liver and skeletal muscle and is known to translocate to the cell surface in response to insulin levels [14, 15] and muscle contraction [16–18]. Several studies have demonstrated that the glucose transport response to insulin in liver and skeletal muscle is greatly integrated to/influenced by glucose transport gene [19, 20].

*Catharanthus roseus* (L.) G. Don (Apocynaceae) is an ornamental shrub, belongs to the family Apocynaceae, is an erected procumbent herb or under shrub containing latex. that grows up to 30–100 cm in height. It was previously known as *Vinca rosea* (L.). it is widely distributed around the World due to its high survivability in a variety of habitats and use as an ornamental plant [21]. *C. roseus* is used traditionally medicine for the treatment of diabetes in several countries of the world [22] including like Nigeria [23], India [23, 24] South Africa, China, Mexico [25] and Malaysia [26]. Roots and leaves of this plant contain more than 100 alkaloids. It has economic importance from its alkaloids. The two leaf alkaloids which are most important in medicine are vinblastine and vincristine [27]. Among few approaches, high performance liquid chromatography (HPLC) technique is still widely used for the separation and analysis of secondary metabolites such as those from *C. roseus* [28]. Fresh leaf juice of *c. roseus* has been reported to reduce blood glucose in diabetic rabbits [24]. Leaf and other part of *c. roseus* also widely used as an infusion for the treatment of diabetes [22] and it has gained acceptance from the pharmaceutical industries [29, 30]. Significant antihyperglycemic activities of the alcoholic extract, [31, 32] aqueous extract, [33] and the dichloromethane-methanol extract of *c. roseus* [34] have been reported in laboratory animals. Although, earlier reports have indicated blood glucose lowering activity in extracts and leaf powder in the management of hyperglycemia have not been undertaken. In view of multidimensional activity of plant drugs, we hypothesized that *c. roseus* leaf would have a complex mechanism of action; therefore, it was imperative to consider different approaches involved in antidiabetic mechanisms. We specifically assumed that insulin secretagogue action might be due to improvement in glucose transport gene (GLUT-2 and GLUT-4) and its effect on transport proteins and fatty acid metabolism.

Therefore, the present study was designed to investigate the insulin mimetic impact of *c. roseus* on the molecular mechanisms of glucose uptake on STZ-induced diabetic rats. With these views, we have studied the effect of *c. roseus* on glucose transport gene messenger RNA (mRNA) expression in the liver STZ model of type 2 DM.

## Methods

### Preparation of plant extract

**Fig. 1:**
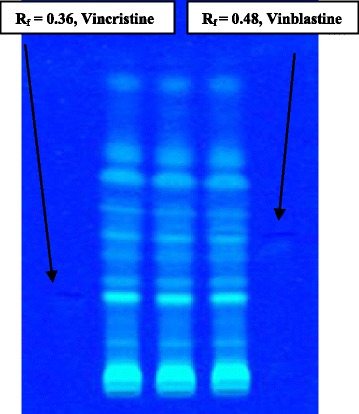
TLC fingerprint of *c. roseus* in triplicate showing at 366 nm

Authentic seeds of *C. roseus* were procured from National Bureau of Plant Genetic Resources (Accessions No., IC 49,595), New Delhi, India. Seeds were grown in the Herbal Garden of Jamia Hamdard, New Delhi, India. Fresh leaf samples were collected and were completely dried under shadow for several days. The dried leaves were grinded into a coarse powder and 100 g powder was mixed well in petroleum ether and kept for 2 days in order to remove all fats, wax and chloroplast etc. After filtration of the material, it was suspended in 500 mL of 95 % ethanol, and the extraction was performed in soxhlet apparatus for 18 h. Then the solvent i.e., ethanol was allowed to evaporate using rotary evaporator at a temperature of 40–45 °C. At the end of the requisite evaporation, we ended up with a highly concentrated ethanolic extract which was filtered using a coarse sieve filter paper. The filtrate was then dried under reduced pressure and finally lyophilized. TLC was run for the ethanolic extract in the solvent system toluene/ethyl acetate/benzene at 6:3:1 ratio and developed in iodine chamber. There were spots clearly observed in all three batches, and Rf values were calculated for standardization parameters in each batch (Fig. 1). The same spots were present in all three batches at the same Rf values. Among few approaches, high performance liquid chromatography (HPLC) technique is still widely used for the separation and analysis of secondary metabolites such as those from *C. roseus* [28]. Extractive values of ethanol soluble, ash value, acid-insoluble ash, and moisture contents were quantified. Phytochemical screening and standardization of the extract were done before commencement of the in vivo study.

### Animals and housing

Specific pathogen-free 30 male wistar rats, weighing 100–150 g and aged 6 weeks were housed in a colony cage (six per cage) (Table 1) at an ambient temperature of 25 °C with 12-h light to 12-h dark cycle. Rats had free access to standard food and water ad libitum. After overnight fasting (deprived of food for 16 h and allowed free access to water), diabetes was induced in rats by intraperitoneally injection of Streptozotocin, dose level of 90 mg/kg dissolved in 1 M freshly prepared sodium citrate buffer pH 4.5 to 2-day-old neonatal rats. The control rats were injected with same amount of citrate buffer. After 6 weeks of injection for the development of diabetes, rats were evaluated for fasting glucose level. The rats with diabetes having glycosuria and hyperglycemia (blood glucose range of above 150–200 mg/dl) were considered as diabetic rats and used for further experiments. The Principles of Laboratory Animal Care (NIH, 1985) were followed throughout the duration of the experiment. The entire animal care and surgery were in accordance with the NIH Guide for the Care and Use of Laboratory Animals (DHEW Publication No. 85–23). Approval to do animal experimentation was obtained from the Institutional Animal Ethics Committee (IAEC) registered under the Committee for the Purpose of Control and Supervision of Experimental Animals (173/CPCSEA).

**Table 1:** Group of animals

S. No.	Group	No of Animals	Description
1.	I	6	Normal Control
2.	II	6	Diabetic Control
3.	III	6	Treated with 100 mg/kg *c. roseus*
4.	IV	6	Treated with 200 mg/kg *croseus*
5.	V	6	Treated with 100 mg/kg Metformin

### Experimental design

A total of 30 rats were weighted before the experiment, and they were divided into 5 groups with 6 rats per group: group 1, normal untreated rats; group 2, diabetic control rats; group 3 and group 4, diabetic rats treated with *c. roseus* 100 mg/kg and 200 mg/kg; and group 5, diabetic rats treated with Metformin (100 mg/kg) (Table 2). After final administration of treatment of 4 weeks, blood samples were collected under fasting conditions, and the body weights (BWs) were measured.

**Table 2:** Effect of *c. roseus* and Metformin on body weight in Streptozotocin-induced diabetic rats

Groups	Treatment	Body weight (g)	
		Initial (g)	Final (g)
I	Normal Control Group	147 ± 3.9	155 ± 3.1
II	Diabetic Group	152 ± 4.1	110 ± 3.3
III	Treated with 100 mg/kg *c. roseus*	138 ± 4.2	144 ± 3.9
IV	Treated with 200 mg/kg *c. roseus*	142 ± 3.4	149 ± 3.2
V	Treated with 100 mg/kg Metformin	137 ± 4.8	148 ± 3.8

### Induction of diabetes

STZ-induced hyperglycemia has been described as a useful experimental model to study the activity of hypoglycemic agents [35]. After overnight fasting (deprived of food for 16 h and allowed free access to water), diabetes was induced in rats by intraperitoneal injection of STZ (Sigma, St. Louis, MO) freshly prepared in 0.1 M sodium citrate buffer, pH4.5, at a dose of 55 mg/kg body weight [31] in five groups of rats. The control rats received the same amount of 0.1 M sodium citrate buffer. After a weeks’ time for the development of diabetes, the rats with diabetes having glycosuria and hyperglycemia (blood glucose range of above 250–300 mg/dl) were considered as diabetic rats and used for further experiments.

### RNA isolation and reverse transcription

Rats were only mildly anesthetized and killed by cervical dislocation. Liver was dissected out and rapidly frozen with tongues cooled to the temperature of liquid nitrogen and stored at −80 °C until analyzed for glucose transport gene mRNA content. Total RNA from liver was extracted with the Qiagen RNEasy Micro kit (GERMANY) including an on-column DNAse I digestion step as described in the manufacturer’s instructions. First-strand complementary DNA (cDNA) was synthesized at 40 °C by priming with oligo-dT12–18 (Invitrogen, USA) and using Super ScriptII reverse transcriptase according to the protocol provided by the manufacturer (Invitrogen, USA).

### Quantitative real time PCR

Real-time polymerase chain reaction (PCR) amplifications for GLUT-4 (gene ID: 25139) were conducted using Light-Cycler 480 (Roche, USA) with the SyBr ® I nucleic acid stain (Invitrogen, USA) according to the manufacturer’s instructions. The cDNA were assayed by duplicate prepared from total RNA using Super Script II (Invitrogen, USA). PCR reactions were performed in a final volume of 25 μl containing 50 ng of templates complementary DNA (cDNA), 2.5 μl of 10× PCR buffer (200 mmol/L Tris, pH 8.4, 500 mmol/L KCl, and 2.5 mmol/L MgCl2), 300 μmol/L deoxyribonucleotides (dNTP), 20 pM of each oligonucleotide, 1:1000 SyBr Green I® nucleic acid stain, 100 μg/ml BSA, 1 % glycerol, 0.5 U *Taq* DNA Polymerase (Invitrogen, USA) final volume maintained with ddH2O. Sets of PCR primers of GLUT-2 sense 5′-CGA CTC GAT CCG TTG GCC TGT CAG CTT GC-3′ and antisense 5′- TGT GTG GAA TTG TCC TCT TAA TCC AGG TCC TG -3′ (Genbank Accession No. NM-012879.2); GLUT-4 sense 5′- TAG AAT TCC CCG GAC CCT ATA CCC TAT TCA TTT-3′ and antisense 5′- TAG GAT CCG CTG TAG AGG AAA GGA GGG AGT CTG -3′ (Genebank Accession No. D28561); and for β-actin sense 5′-TCA CCC ACA CTG TGC CCC ATC TAC GA-3′ and antisense 5′ CAG CGG AAC CGC TCA TTG CCA ATG G-3′ (Genebank Accession No. NM031144.2) were designed with reference to the National Centre for Biotechnology Information (NCBI) database of conserved coding regions. Thermal cycling conditions were performed 95 °C for a 2.5 min initial denaturation step followed by 40 PCR cycles (94 °C for 20 s, 58 °C for 30 s, and 72 °C for 50 s). Fluorescence was detected at the end of the 58 °C segment. Polymerase chain reaction products of β-actin primer gene were used as an internal standard. Amplifications were performed in duplicate for the entire samples. Real-time PCR analysis and subsequent calculations were performed on Light-Cycler 480 software (version LCS480 1.2.0.169; Roche). The relative starting quantity of each transcript was determined using the comparative C_T_ method for relative quantification [36].

### Statistical analysis

The results are expressed as mean ± SD. Statistical evaluation was carried out using ANOVA followed by Dunnett’s test between the groups. Differences were considered significant at *p* < 0.05 and highly significant at *p* < 0.01.

## Results

**Fig. 2:**
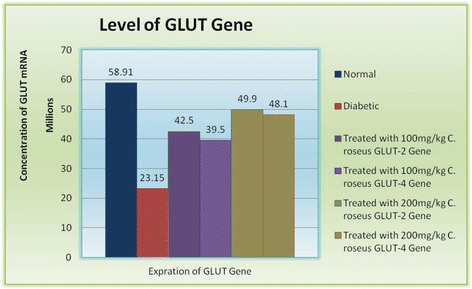
Effect of *c. roseus* on GLUT-2 & GLUT-4 mRNA level with deferent concentration 100 mg/kg on GLUT-2, 100 mg/kg on GLUT-4, 200 mg/kg on GLUT-2 and 200 mg/kg on GLUT-4

In Diabetic rats treated with ethanolic extract of *c. roseus* leaf and metformin, there were no significant differences of baseline body weight of the rats. The rats treated with *c. roseus* 100 mg/kg, 200 mg/kg and metformin 100 mg/kg showed no significant increase in body weight as compared with diabetic groups after 4 weeks of study. Blood glucose level of every 2 day (0.1–0.2 ml blood collected from tail) interval showed significance with treatment of *c. roseus* to diabetic animal group in comparison to metformin-treated group for 20 days (Table 3). *C. roseus* (100 mg/kg BW) lowered the glucose level than metformin-treated group (100 mg/kg BW) (Table 4). *C. roseus* 200 mg/kg dose was found to be more effective in reducing fasting blood glucose levels Fig. 2.

**Table 3:** Effect of ethanolic extract of *c. roseus* on some biochemical parameter on rats

Dose mg/kg	GLU mg/dl	CHOL mg/dl	CRE Umol/L	ALP IU/L	ALT IU/L	AST IU/L	GST IU/L	BUN mg/dl
Normal Control Group	98.9 ± 2.28	59.12 ± 5.08	107.24 ± 4.08	102.4 ± 3.81	56.82 ± 5.04	87.28 ± 5.83	0.59 ± 0.05	38.40 ± 2.36
Diabetic Control Group	256.32 ± 8.64	117.34 ± 2.20	196.74 ± 2.84	286.21 ± 5.34	134.20 ± 7.41	37.43 ± 4.59	0.45 ± 0.06	116.2 ± 1.24
Treated with 100 mg/kg *C. roseus*	189.42 ± 11.2*	92.49 ± 7.17*	168.26 ± 6.23*	185.36 ± 4.32*	98.64 ± 8.80*	116.16 ± 4.51*	0.44 ± 0.07	86.26 ± 2.49*
Treated with 200 mg/kg *C. roseus*	153.34 ± 6.72**	85.43 ± 6.61**	132.41 ± 6.31**	150.42 ± 4.78**	81.38 ± 4.86**	125.29 ± 5.09	0.44 ± 0.07	65.41 ± 5.23**
Treated with 100 mg/kg Metformin	135.56 ± 9.72**	68.04 ± 5.80**	127.74 ± 3.16**	126.46 ± 3.68**	76.44 ± 4.72**	129.52 ± 3.96**	0.44 ± 0.07	45.72 ± 4.84**

Results are mean ± SD, n05

*GLU* glucose, *CHOL* cholesterol, *CRE* creatinine, *ALP* alkaline phosphatase, *ALT* alanine transaminase, *AST* aspartate transaminase, *GST* Glutathione *S*-transferase, *BUN* blood urea nitrogen

Values are expressed as mean ± SD for six animals in each group. *Level of significance p < 0.05, **Level of significance p < 0.01

**Table 4:** Effect of ethanolic extract of *c. roseus* on blood sugar at different time intervals in steptozotocin-induced diabetic rats

Groups	Blood glucose (mg/dl)								
	Before treatment	After treatment							
		8^th^ day	11^th^ day	14^th^ day	17^th^ day	20^th^ day	23^th^ day	26^th^ day	28^th^ day
Normal Control Group	113 ± 3.5	107 ± 3.4	109 ± 3.9	115 ± 4.2	112 ± 4.3	108 ± 3.3	109 ± 2.9	105 ± 3.3	110 ± 2.9
Diabetic Group	112 ± 4.4	248 ± 3.8	281 ± 3.1	299 ± 3.9	314 ± 3.1	329 ± 3.6	343 ± 3.5	373 ± 3.1	398 ± 3.4
Treated with 100 mg/kg *c. roseus*	118 ± 4.8	254 ± 3.1	242 ± 4.3	235 ± 4.7	224 ± 4.0	212 ± 3.7	195 ± 4.3	180 ± 4.1	138 ± 4.8
Treated with 200 mg/kg *c. roseus*	114 ± 3.9	269 ± 3.9	232 ± 4.1	223 ± 3.6	218 ± 4.4	205 ± 4.7	190 ± 3.3	169 ± 4.5	123 ± 4.1
Treated with 100 mg/kg Metformin	117 ± 4.1	247 ± 3.2	243 ± 3.6	238 ± 3.1	225 ± 3.6	214 ± 3.6	201 ± 3.9	188 ± 3.6	141 ± 3.5

RNA concentrations were measured from liver samples. It was therefore necessary to confirm that the control gene for quantitative real time PCR was not over-represented. We measured transcription of the control β-actin in cDNA prepared from these samples, quantified it using the standard curve method. As shown in Fig. 2, the amplification efficiency (E) for genes was determined by linear regression analysis of the fluorescent data from the exponential phase of PCR [37]. Quantitative reverse transcription RT-PCR results showed increase in liver GLUT-2 mRNA and GLUT-4 mRNA concentrations after 4 weeks treatment of *c. roses* as compared with corresponding vehicle-treated diabetic rats.

**Fig. 3:**
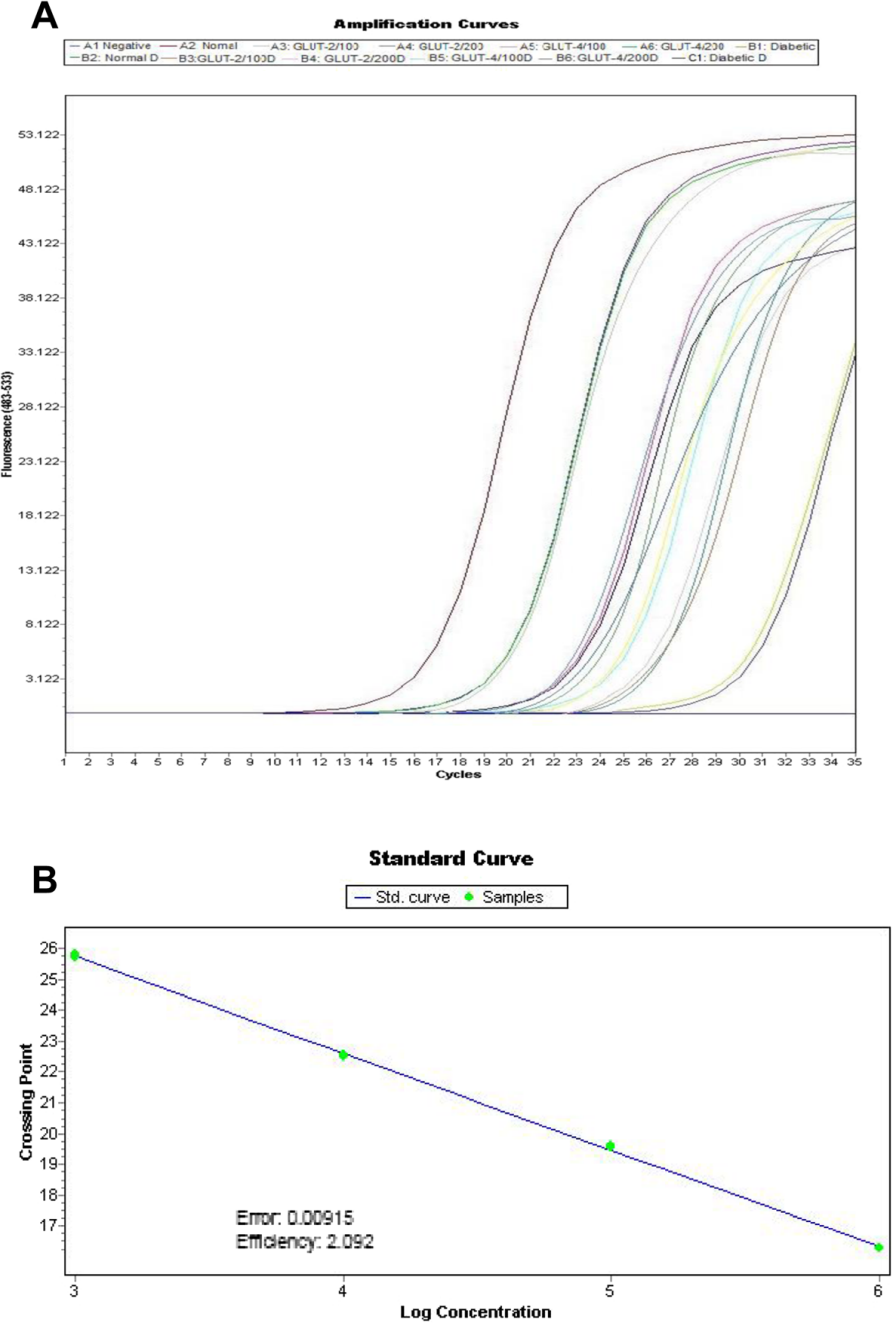
Simultaneous amplification plots and standard curves of GLUT-2 & 4 mRNA by fluorescence real-time PCR. **a**. Plot of threshold cycle number (Ct) vs. serial dilutions (log10) of standard DNA from 10^1^ to 10^8^ copies/reaction tube were prepared. Reaction number increases during PCR as the amplicon copy number increases until the reaction reaches a plateau. Ct was plotted against each copy number. Ct represents the PCR cycle at which reporter signal can first be detected. **b**. Standard curve showing efficiency of amplification during PCR

Because there was an increase in plasma insulin levels in *c. roseus* treated diabetic rats and because of the physiologic importance of insulin-dependent glucose transport gene (GLUT-2 and GLUT-4) translocation to the cell membrane, attempts have been made to see the effect of *c. roseus* treatment on liver tissue GLUT-2 and GLUT-4 levels. In the liver and muscle membrane fractions of diabetic rats, the translocation of GLUT-2 and GLUT-4 was very much reduced when compared with the band density of healthy controls. This is quite rational because the deficiency of insulin in the diabetic state would decrease the translocation of GLUT-2 and GLUT-4 from the vesicles to cell membranes. Treatment with *c. roseus* resulted in the reversal of membrane GLUT-2 and GLUT-4 levels. The glucose transport gene (GLUT-2 and GLUT-4) levels of normal control, diabetic, and *c. roseus* -treated diabetic rats are shown in Fig. 3.

## Discussion

Diabetes mellitus is an endocrine and metabolic disorder indicated by chronic hyperglycemia that produces biochemical changes and tissue destruction. Several obstacles of biochemical changes and tissue destruction like atherosclerosis, neuropathy, nephropathy, etc. have chronically elevated glucose levels due to glycosylation and metabolic disorder. Diabetic stress and other biological changes in tissue have been reduced by antioxidants [38]. STZ induced hyperglycemia in rodents is considered to be a good preliminary screening model [39] and is widely used. STZ is a potent methylating agent for DNA and acts as nitric oxide donor in pancreatic cells. Literature for synthetic drugs for diabetes mellitus tells that most of these drugs have many side effects. So, scientists are in search of safe, natural anti-diabetic agents that can cure the diabetes without causing harm, and the World Health Organization has also recommended the development of herbal medicine in this concern [40].

In the present study, a significant blood glucose reduction was observed in healthy rats after the intraperitoneal administration of ethanolic extract of *c. roseus* leaf 100 mg/kg and 200 mg/kg and metformin 100 mg/kg in a preclinical model of STZ-induced type 2 diabetes for period of 4 weeks. The body weight of diabetic rats was found to be less during the course of the development, which may be because of accelerated lipolysis, whereas weight gain was significantly observed in rats treated for 4 weeks with *c. roseus* treated and metformin. Significant changes are observed after *c. roseus* treatment in the distribution of insulin, glucagon, and glucose transport gene (GLUT-2 and GLUT-4). The level of glucose transport gene increases markedly in the *c. roseus* treated sample is much greater in the untreated rats when compared with control animals. The glucose transport gene expression is down-regulated when there is relative insulin deficiency, such as in STZ-induced diabetes [41].

After treatment with *c. roseus*, the contents of glucose transport gene mRNA were restored to near normal values. The decrease in glucose transport gene levels is essentially one of the main reasons of hyperglycemia in the diabetic state, which is due to decreased uptake of glucose. Restoration of glucose transport gene levels would, therefore, enhance the uptake of glucose in liver and thus help to combat hyperglycemic conditions. Glucose unresponsiveness associated with GLUT gene impairment is typically demonstrated in type 2 diabetes [42].

## Conclusion

In conclusion, the *c. roseus* plant ethanol extract was found to exhibit a significant anti hyperglycemic activity in STZ-induced diabetic rats. The results from blood glucose, serum biochemical estimation and glucose transport gene (GLUT-2 and GLUT-4) mRNA indicated that the *c. roseus* has renewing and healing ingredients as it could reverse most blood and tissue changes caused by STZ-induced diabetes in rats.
